# Refining the time–frequency characteristic of non-stationary signal for improving time–frequency representation under variable speeds

**DOI:** 10.1038/s41598-023-32333-w

**Published:** 2023-03-30

**Authors:** Yi Liu, Hang Xiang, Zhansi Jiang, Jiawei Xiang

**Affiliations:** 1 College of Mechanical and Electrical Engineering, Wenzhou University, Wenzhou, 325035 People’s Republic of China; 2 School of Mechanical and Electrical Engineering, Guilin University of Electronic Technology, Guilin, 541004 People’s Republic of China; 3 School of Mathematics and Computer Science, Northwest Minzu University, Lanzhou, 730000 People’s Republic of China; 4 Pingyang Institute of Intelligent Manufacturing, Wenzhou University, Wenzhou, People’s Republic of China

**Keywords:** Engineering, Mathematics and computing

## Abstract

Time–frequency ridge not only exhibits the variable process of non-stationary signal with time changing but also provides the information of signal synchronous or non-synchronous components for subsequent detection research. Consequently, the key is to decrease the error between real and estimated ridge in the time–frequency domain for accurate detection. In this article, an adaptive weighted smooth model is presented as a post-processing tool to refine the time–frequency ridge which is based on the coarse estimated time–frequency ridge using newly emerging time–frequency methods. Firstly, the coarse ridge is estimated by using multi-synchrosqueezing transform for vibration signal under variable speed conditions. Secondly, an adaptive weighted method is applied to enhance the large time–frequency energy value location of the estimated ridge. Then, the reasonable smooth regularization parameter associated with the vibration signal is constructed. Thirdly, the majorization–minimization method is developed for solving the adaptive weighted smooth model. Finally, the refined time–frequency characteristic is obtained by utilizing the stop criterion of the optimization model. Simulation and experimental signals are given to validate the performance of the proposed method by average absolute errors. Compared with other methods, the proposed method has the highest performance in refinement accuracy.

## Introduction

Time–frequency analysis (TFA) method is an effective tool to provide information on signal synchronous or non-synchronous components in condition monitoring and fault diagnosis under non-stationary conditions. Furthermore, the time-varying features of non-stationary signals could be characterized. TFA methods are wildly applied in radar, sonar and astronomical, biomedicine, and mechanical engineering areas^1–6^, etc. The conventional TFA methods are roughly divided into linear and quadratic transforms, and all of them have respective drawbacks. For example, short-time Fourier transform (STFT) and continuous wavelet transform (CWT) and so on, both of which are difficult in choosing a reasonable window parameter of TFA, which leads to time and frequency resolution in the time–frequency domain^7^. On the other hand, the classical quadratic transform represented by Wigner–Ville distribution (WVD), the cross-term interferences would be introduced in analyzing multi-component signal^8^, which decreases the readability of time–frequency, and increases the difficulty of time–frequency ridge extraction.

Mostly, the peak value search algorithm is always applied to extract the peak energy of time–frequency representation for characterizing the procedure of time-varying signal in the industry area. Nevertheless, the obtained peak ridge is a rough curve using the aforementioned time–frequency methods. Therefore, the rough curve is an approximated broken line although constructing a suitable window parameter.

To mitigate the impact of entangled background noises and interferences in analyzing time-varying signals and obtain concentrated time–frequency representation, the post-progressing tool is introduced to solve the above problems. Auger^9,10^ proposed a reassignment (RM) technique to concentrate the time–frequency energy into a narrow band. After that, the synchrosqueezing transform (SST)^11^ is proposed to squeeze the time–frequency coefficients into the instantaneous frequency (IF) trajectory along the frequency axis, the method could provide fine time–frequency readability. In other words, the blurry time–frequency representation is concentrated by using a synchrosqueezing operator when analyzing a stationary signal, as a result, an accurate time–frequency representation is obtained^12^. Nevertheless, the fitted time–frequency curve is heavily biased in comparison with the real IF when analyzing chirp signals or frequency-modulated signals^13,14^. Several years ago, Yang proposed a series of parametric time–frequency analysis methods to characterize the variety of the time-varying signal^15–17^. It is worth mentioning that author extended the conventional linear chirp kernel to a polynomial chirplet transform (PCT) by constructing a polynomial nonlinear chirplet kernel to replace the chirplet kernel in the chirplet transform. In the same way, spline-kernelled chirplet transform (SCT) is developed. (Weierstrass approximation theorem is applied to guarantee that any continuous function on a closed and bounded interval can be uniformly approximated on that interval by a polynomial to any degree of accuracy, however, the order value should be determined in advance^15^). Although the time–frequency trajectory of a time-varying signal is well-fitted, the time–frequency representation energy is blurry. In recent years, some useful improved techniques is proposed to process non-stationary signals, second-order STFT-based SST (FSST2)^18^ and high-order SST^19^ are developed to match amplitude modulation (AM) and frequency modulation (FM) multi-component signals^20^, meanwhile, the time–frequency energy is concentrated into a narrow band. However, the complexity and diversity of practical cases are difficult to determine the accurate parameters of IF^17,21^. Yu proposed an iterative technique to improve the time–frequency energy concentration compared with the SST method, iterative technique not only processes time-varying signals but also has been validated in the advantage of concentrating energy by computing the index of Rényi entropy^22^. Although the time–frequency readability is obtained by introducing a high-order synchrosqueezing operator and iterative techniques, the estimated time–frequency trajectory is broken-line.

The smoothing technique is wildly applied in scientific research and industry areas. The sampled data are always affected by vibration, electromagnetic interference, transmission path, quantization error, and so on; consequently, the obtained data is mutational, with spikes, and jump^23–25^. Therefore, it is important to confirm the obtained data is reliable and available before signal processing. Aimed at solving the problem of broken-line for extracting time–frequency trajectory and then to refine the rough curve for obtaining a more accurate curve. Firstly, Yang applied by PCT or SCT method to obtain IF trajectory^16,26^, secondly, searching the peak values of time–frequency representation and then fitted it, and finally, smoothed the rough curve by the least square method (LSM) to obtain more accuracy estimated curve. Nevertheless, if the feature matrix is non-invertible or ill-conditioned, the analytical solution of LSM cannot be obtained. Non-invertible means that the data is linear correlation and redundancy. For an ill-conditioned matrix, the obtained analytical solution is sensitive to little change in a coefficient matrix or constant term. Therefore, the regularization term is added to the optimal function to avoid the aforementioned problems. The most famous methods named ridge regression, least absolute shrinkage, and selection operator (LASSO). The regularization term of ridge regression is L2-norm which is differentiable. Nevertheless, the super-parameter selection is a great important problem in a ridge regression model. In 2017, Chen proposed a method that formulates an optimal demodulation problem to construct a time–frequency filter bank for obtaining a narrow-band signal^27^. The author applied a ridge regression model to smooth the time–frequency curve, the smaller penalty parameter is constructed to ensure a smoother time–frequency trajectory^28^. L1-norm is applied in the LASSO model, which could select an argument and squeeze the coefficient of the negligible argument in zero value. Therefore, the LASSO model also called the smooth model in optimization fields and it is a perfect tool to de-noise vibration signals. L1-norm could not differentiable at zero point and the obtained solution is not analytical. Sometimes, the regularization parameter is always set constant value instead of changing with the signal, and L1-norm and L2-norm are applied to avoid non-invertible and sensitivity to little change of coefficient matrix or constant term in the LSM method.

Therefore, in this article, an adaptive weighted smooth model (AWMM) is proposed to solve the aforementioned problems. The regularization parameter associated with the vibration signal is constructed, which does not depend on any prior knowledge of the tested signal. Furthermore, the prior regularization parameter can be determined by the signal itself. Majorization–minimization (MM) method is introduced to solve the problem of non-differentiable at zero point. Based on the estimated coarse time–frequency ridge by the multi-synchrosqueezing transform (MSST) method^22^, the ridge is smoothed and then achieves high accuracy using AWMM. The proposed model not only could eliminate the unrelated components of the estimated coarse IF but also provide the refined IF accurately.

This article is organized as follows: the theoretical background of MSST and AWMM is displayed in “Method”. The completed refine procedure of the vibration sigal is shown in “Numerical simulations”. In “Experiment investigation”, the performance of the proposed method is validated by simulation and experimental signals. Finally, the conclusion is shown in “Conclusion”.

## Method

Inspired by the formula of IF smooth construction in^27^, the constructed model could be further to be improved, because the key penalty parameter of the model is difficult to be determined. In this section, a signal-driven technique is introduced to solve the above problem and the model is enhanced to improve the IF accuracy in linear and nonlinear time-varying conditions. The common optimal models are used to eliminate the unconcern components of signals and make errors between the estimated and the actual values decrease, for example, LASSO and ridge regression et al. To convenient to express the above two methods, the former called L1-based optimal function and the latter named L2-based optimal function. 

The smooth model is constructed as follows:

1 $$ F(f) = \frac{1}{2}\left\| {f - \tilde{f}} \right\|_{2}^{2} + \lambda \left\| {Df} \right\|_{1} $$

where the $\tilde{f}$ is the calculated coarse IF of the signal, the estimated IF could be a nonlinear curve, thus $\tilde{f} = [\tilde{f}(t_{0} ),\tilde{f}(t_{1} ),...,\tilde{f}(t_{N - 1} )]$, and the $f$ is the corresponding refined IF, $f = [f(t_{0} ),f(t_{1} ),...,f(t_{N - 1} )]$. The constructed model can refer to^27,29^. To decrease the end effects caused by the difference operation, the second-order difference matrix is given as $${\mathbf{D}} = \left[ {\begin{array}{*{20}c} { - 1} & {2} & {\begin{array}{*{20}c} { - 1} & {} & {} \\ \end{array} } & {} \\ {} & { - 1} & {\begin{array}{*{20}c} {2} & { - 1} & {} \\ \end{array} } & {} \\ {} & {} & {\begin{array}{*{20}c} {} & {...} & {} \\ \end{array} } & {} \\ {} & {} & {\begin{array}{*{20}c} {} & { - 1} & 2 \\ \end{array} } & { - 1} \\ \end{array} } \right]$$ , the size of the matrix $(N - 2) \times N$, and the N has defined as the length of $f$ and $\lambda$ is the regularization parameter. It is important to set a suitable $\lambda$ initially, in the subsequent section, the rule of the determined parameter would be given. The penalty term of the proposed model is to let the coefficients of the signal approximate zero or equal to zero and further eliminate the unrelated components of the signal. Sometimes, the regularization parameter is always set constant value instead of changing with the signal and it is obvious that the same parameter corresponding to each point is unsuitable. Therefore, an adaptive weighted technique is introduced to address the above problem, and then the initial regularization parameter is determined by the signal. The corresponding formula of the initial value is set

2 $$ \lambda_{0} = \frac{{\left\| {\tilde{f}} \right\|_{2} }}{N} $$

and the formula of adaptive weighted is given

3 $$ w_{j} = \frac{1}{{x_{j - 1} + \varepsilon }} (\varepsilon = 1e - 6)$$

where the *j* is the iteration count and the value is from 1 to J, when $j = 1$, the weight matrix could be a unit matrix *I*, $W = diag(w_{1} ,w_{2} ,...,w_{N - 2} )$, the size of this matrix is $(N - 2) \times (N - 2)$. Equation (1) could be rewritten as Eq. (4).

4 $$ F(f) = \frac{1}{2}\left\| {f - \tilde{f}} \right\|_{2}^{2} + \left\| {W^{\prime}f} \right\|_{1} $$

To express the penalty term briefly, the $W^{\prime} = \lambda_{0} WD$, thus, the refined IF $f$ could be calculated as

5 $$ \hat{f} = \mathop {\arg \min }\limits_{f} F(f) $$

Considering that the conventional penalty function is non-differentiable at zero point, the majorization–minimization (MM) algorithm is applied to realize non-differential elimination at zero point, the pivotal of the MM algorithm is to seek a majorizer $G(f,u)$ of $F(f)$, and the majorizer $g(f,u)$ of $\varphi (f)$, ($\varphi (f) = \left\| {W^{\prime}f} \right\|_{1}$), they must meet the following formula:

6 $$ g(f,u) = mf^{2} + b $$

7 $$ g(f,u) \ge \varphi (f) $$

8 $$ g(u,u) = \varphi (f) $$

where the two variables satisfy the condition $f,u \in R$, after calculating Eqs. (7) and (8), the obtained equations are as follows

9 $$ g(u,u) = \varphi (u) = mu^{2} + b $$

10 $$ g^{\prime}(u,u) = \varphi^{\prime}(u) = 2mu $$

in which the unknown variables *m* and *b* could be solved by the method of undetermined coefficients (MUC), the obtained equations are shown

11 $$ m = \frac{{\varphi^{\prime}(u)}}{2u} $$

12 $$ b = \varphi (u) - \frac{u}{2}\varphi^{\prime}(u) $$

Therefore, the majorizer function is detailed to represent in Eq. (13)

13 $$ g(f,u) = \frac{{\varphi^{\prime}(u)}}{2u}f^{2} + \varphi (u) - \frac{u}{2}\varphi^{\prime}(u) $$

It is noted that the L1 norm of the optimal model can be defined as

14 $$ \left\| f \right\|_{1} = \sum\limits_{n = 0}^{N - 1} {\left| {f_{n} } \right|} $$

therefore, Eq. (13) can be revised as

15 $$ \sum\limits_{n = 0}^{N - 1} {g(f_{n} ,u_{n} )} = \frac{1}{2}f^{T} [\Lambda (u)]f + b(u) \ge \sum\limits_{n = 0}^{N - 1} {\varphi (f_{n} )} $$

where the diagonal matrix is represented $[\Lambda (u)] = diag(\frac{{\varphi^{\prime}(u_{n} )}}{{u_{n} }})$ and scalar $b(u) = \sum\nolimits_{n = 0}^{N - 1} {[\varphi (u_{n} ) - \frac{1}{2}\varphi^{\prime}(u_{n} )} ]$. When we considered the penalty term of Eq. (4), Eq. (15) would be revised

16 $$ \frac{1}{2}(W^{\prime}f)^{T} [\Lambda (W^{\prime}u)]W^{\prime}f + b(W^{\prime}u) \ge \left\| {W^{\prime}f} \right\|_{1} $$

when the equation $f = u$, the majorizer function $G(f,u)$ is given

17 $$ G(f,u) = \frac{1}{2}\left\| {f - \tilde{f}} \right\|_{2}^{2} + \frac{1}{2}(W^{\prime}f)^{T} [\Lambda (W^{\prime}u)]W^{\prime}f + b(W^{\prime}u) $$

this is a minimized problem and its analytical solution could be obtained

18 $$ \hat{f} = [I + (W^{\prime})^{T} [\Lambda (W^{\prime}u)]W^{\prime}f]^{ - 1} \tilde{f} $$

The proposed model not only could eliminate the unrelated components of the estimated coarse IF but also provide the refined IF accurately. The important parameters of the model can be determined adaptively based on the signal itself.

## Numerical simulations

In this section, linear and non-linear simulated signals are used to demonstrate the capability of the AWMM to smooth the time–frequency curves. We focus on the comparisons between the AWMM method and other common smooth techniques in addressing linear and nonlinear signals. The comparisons mainly focus on the smooth accuracy between the real IF curve and the post-processed curve. Due to the mean absolute error (MAE) does not appear positive and negative off settings in the assessment of the error of estimated and real values, this index is introduced in this article to measure the performance of the proposed method. The absolute is a mathematical function that makes a number positive. The obtained MAE value is less than 1. Especially, the MAE value will no longer be calculated if the calculation results of the comparison method are too different. The sampling frequency is 100 Hz. It is necessary to compare similar results to test the performance of the mentioned methods. The refined and real curves differ greatly, and the calculated index value of MAE is meaningless. Considering that the experimental data provide the coarse curves, we develop the comparison cases of time–frequency analysis methods in simulation parts. We use traditional and enhanced time–frequency analysis methods to check the performance of AWSM, such as CWT and SST. CWT is a multi-resolution analysis method, which can process stationary and non-stationary signal well. SST can concentrate time–frequency energy into a limit band for separating signal’s components.

Herein, a linear simulated signal is modeled and its corresponding IF is given

19 $$ s = \sin (2\pi (40t - t^{2} )) $$

20 $$ {\text{IF}} = 40 - 2t $$

where the time duration is 4 s. The obtained results of the L2-based and L1-based optimal functions, the proposed method, and polynomial curve fitting-based LSM are exhibited in Fig. S1, all results are presented in the Supplementary information document, namely “All computed compared results.pdf”.

**Figure 1:**
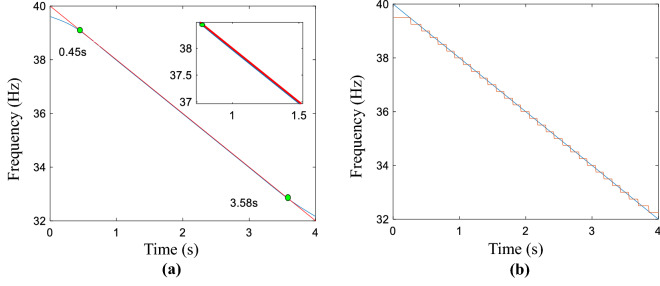
Simulated signal. (**a**) Obtained result by using the proposed method, (**b**) the estimated and real curves.

The smooth result generated by the proposed method is given in Fig. 1a, which matches the point from 0.45 to 3.58 s, the obtained fitting region is the biggest for all the mentioned methods. That is 3.13 s. The performance of the proposed method is verified by the calculated fitting region. The real and estimated curves is displayed in Fig. 1b, which the red presents coarse IF and blue is defined as real IF. The coarse IF is extracted from time–frequency plane using MSST method. As a consequence, the matched region results of the refined line by using the above methods are given, the AWMM method could match most of the IF trajectory. The accuracy level of the above method is testified by calculating the index of MAE, the calculated result is 0.0411, which is smaller than the MAE of Fig. 1b. The MAE of real and estimated curves is 0.0791. To a certain extent, the proposed method could improve the accuracy of the refined curve. Furthermore, the calculated the index values of all methods are demonstrated in Table S1, which is in the Supplementary information document, namely “All computed compared results.pdf”. The results of SST and CWT methods are displayed in Fig. S2, meanwhile, the corresponding smooth results is filled in Table S2, which is in the Supplementary information document, namely “All computed compared results.pdf”.

The sampled time is 6.5 s and the simulated non-linear signal is as follows:

21 $$ s = \sin (2\pi (30t - 6\sin (1.2t))) $$

22 $$ {\text{IF}} = 30 - 7.2\cos (1.2t) $$

**Figure 2:**
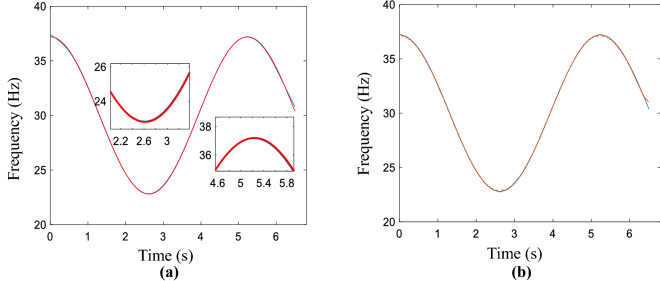
Simulated signal. (**a**) Obtained result by using the proposed method, (**b**) the estimated and real curves.

This test is to consider the performance of the AWMM method in smoothing the sinusoidal signal. In the same way, the estimated coarse curve is smoothed by the penalty function, the proposed method, and the LSM method, and the corresponding results are given in Fig. S2, all results are presented in the Supplementary information document, namely “All computed compared results.pdf”. Similarly, the red line is the real IF curve and the blue one is the smooth curve. In the non-linear time–frequency ridge refinement case, the enlarged locations are the peak and trough of the rough curve. The calculated result by using the AWMM method is shown in Fig. 2a. No matter the peak or trough location, the refined curve is fitted accurately. Compare with Fig. 2b, that consists of real and estimated curves, the calculated MAE value is 0.0553, which is bigger than the value of the AWMM method. Most points are matched with the red line and the MAEs of the aforementioned methods are calculated as in Table S2, which presents in the Supplementary information document. The minimum value belongs to the method proposed in this section and provides the refinement IF with the highest accuracy in nonlinear time-varying conditions. On the other hand, the performance of the MSST method is validated by comparing SST and CWT methods, the results are shown in Fig. S4 and Table S4, they are presented in the Supplementary information document.

The calculated the index values of all methods are demonstrated in Table S2 and S2, from the tables, although the MAE value of the proposed model is not the smallest in linear case, the most curve trajectory is tracked by comparing with LSM and L2-based model. Both of LSM, L2-based model and AWSM method all have very close value.

In nonlinear case, AWSM has the smallest MAE value by comparing with the other methods, furthermore, the nonlinear operation environments are frequently in practical application. Therefore, the performance of the proposed model can be checked by the two cases.

## Experiment investigation

**Figure 3:**
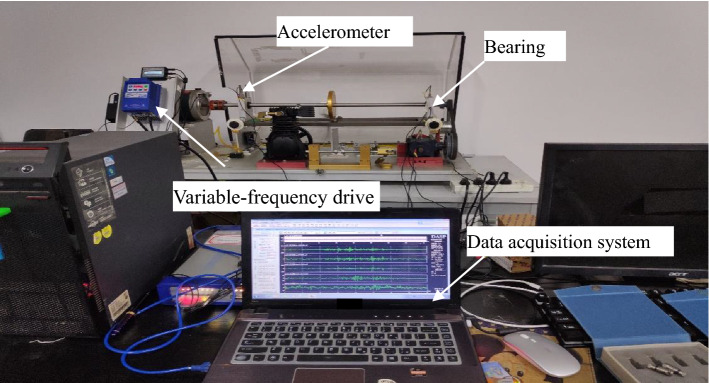
The MFS-MG test rig.

In this section, the proposed method is further tested by a rolling bearing under non-stationary conditions, such as linear time-varying, and nonlinear time-varying. The collected signals are from the Guilin University of Electronic Technology lab and the types of experimental bearing are ER-12K and ER-16K. The experiments were conducted on the machinery fault simulator test rig of SpectraQuest Co, which is shown in Fig. 3. Two accelerometers are installed on the rolling bearing in vertical and parallel directions, respectively.

**Figure 4:**
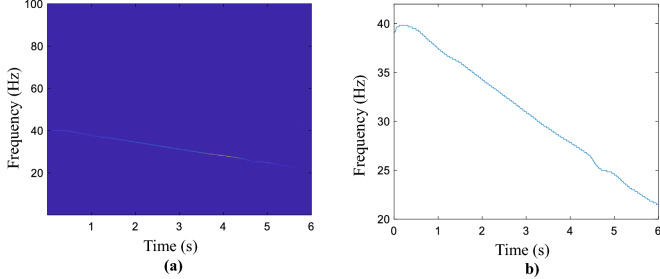
Linear time-varying vibration signal. (**a**) Obtained time–frequency representation of the signal by using MSST, (**b**) corresponding coarse estimated IF.

**Figure 5:**
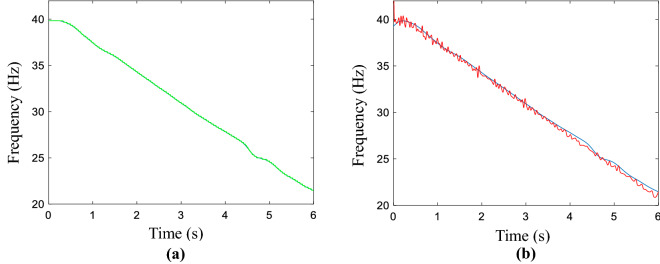
Results of the refined IF. (**a**) The estimated and refined result, (**b**) the real and refined result.

In this subsection, the linear time-varying vibration signal is collected to testify to the performance of the proposed smooth method. The sampling frequency is set at 25.6 kHz and the sampled signal length is 12.8 s. To improve the computation efficiency, we select 153,600 samples as the tested signal. Meanwhile, the key-phase signal is recorded by a tachometer, and then the real IF is calculated for comparison, furthermore, due to the calculation method and other reasons, the real IF obtained is not smooth. The time–frequency representation conducted by the MSST method, as is shown in Fig. 4a, and the corresponding rough IF curve is displayed in Fig. 4b, the broken line is presented by magnifying the IF trajectory. Figure S5 presented the IF refinement results of L2-based and L1-based optimal functions, and the LSM method. The obtained results are presented in the Supplementary information document, namely “All computed compared results.pdf”. In Fig. 5a, the green line is the estimated line and the blue line is the refined IF curve. The green line is surrounded by the green line and it’s a smooth line. From Fig. 5b, the refined curve is close to the real IF and track the variable tendency. The compared results are displayed in Fig. S6, and the MAEs of the aforementioned methods are calculated as in Table S5, which could refer to the Supplementary information document, namely “All computed compared results.pdf”.

**Figure 6:**
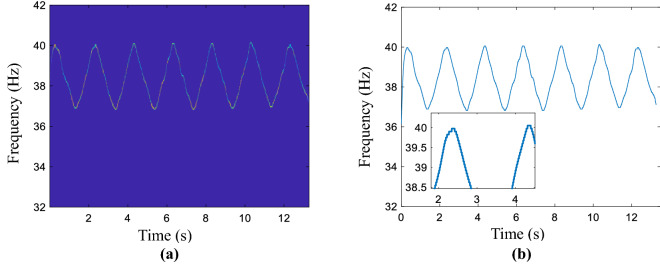
Non-linear time-varying vibration signal. (**a**) Obtained time–frequency representation of the signal by using MSST, (**b**) corresponding coarse estimated IF.

The IF of the vibration signal is an important indicator for the condition monitoring of rotating machinery, especially in complex operating conditions. In this section, the real IF of the collected signal is up and down 2 Hz fluctuations, and the baseline is 38 Hz. The sampling frequency is 12.8 kHz and the signal length is 13.28 s. The generated by the MSST method is shown in Fig. 6a and it's the estimated IF is given in Fig. 6b. Fig. S7 shows the IF refinement results of the vibration signal by using L2-based and L1-based optimal functions, and the LSM method. The refined and real results are exhibited in Fig. S8. Both of them are presented in the Supplementary information document, namely “All computed compared results.pdf”.

**Figure 7:**
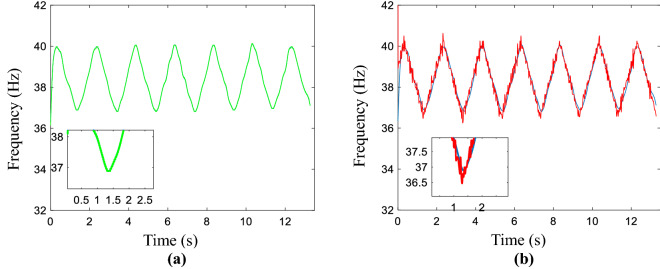
Results of the refined IF. (**a**) The estimated and refined result, (**b**) the real and refined result.

The green line is the estimated line and the blue line is the refined IF curve. In Fig. 7a, the broken line not only is smoothed but also infinitely close to the estimated line using the AWMM method. Similarly, the blue line is the real IF curve and the red line is the estimated IF curve. From Fig. 7b, the fitting effects are more accurate than the mentioned methods, which are provided by the proposed method. the MAEs of the aforementioned methods are calculated as in Table S6, which could refer to the Supplementary information document, namely “All computed compared results.pdf”.

## Conclusion

In this article, an adaptive weighted smooth model for smoothing ridge and improving estimation accuracy is developed. An adaptive weighted method is utilized to enhance the large energy value location of the estimated ridge. The regularization parameter is determined by the signal automatically. Meanwhile, the MM-based iterative method is employed to solve the construction convex model. Based on the estimated coarse time–frequency ridge by MSST computation, the ridge is smoothed to achieve high accuracy using AWMM. Thereafter, the index of MAE values is adopted to check the performance of the proposed method. The numerical and physical experiments are performed and the results show that the proposed method is more accurate than the commonly used polynomial curve fitting-based LSM method and L2-based norm regularization method. Moreover, the proposed method is superior to L1-based norm regularization with the same regularization parameter. Nevertheless, the proposed method has the main drawback to process fast time-varying signals. Future work can mainly consider developing the general refined method and expanding the proposed method application in multiple work conditions.

## Supplementary Information

Supplementary Information 1.

Supplementary Information 2.

## Data Availability

The datasets generated during and/or analyzed during the current study are available from the corresponding author on reasonable request.
