# Supplementary material for: Refining the time–frequency characteristic of non-stationary signal for improving time–frequency representation under variable speeds
Source: Sci Rep. 2023 Mar 30;13:5215. doi: 10.1038/s41598-023-32333-w (PMC10063649; doi:10.1038/s41598-023-32333-w)
Supplement: Supplementary file 1 — Supplementary Information 1. [file 41598_2023_32333_MOESM1_ESM.pdf]

Supplementary Information:” All computed compared results”

This document is supplementary information for the paper entitled “Refining the time-frequency characteristic of non-stationary signal for improving time-frequency representation under variable speeds”, authored by Yi Liu et al.

To demonstrate the performance of the smooth model in refining linear and non-linear curves, three smooth methods are introduced to process the estimated coarse curves. Due to the mean absolute error (MAE) does not appear positive and negative off settings in the assessment of the error of estimated and real values, this index is introduced in this article to measure the performance of the proposed method. The obtained MAE value is less than 1. Thus, the accuracy of the refined curves can be measured from the index value. Especially, the MAE value will no longer be calculated if the calculation results of the comparison method are too different.

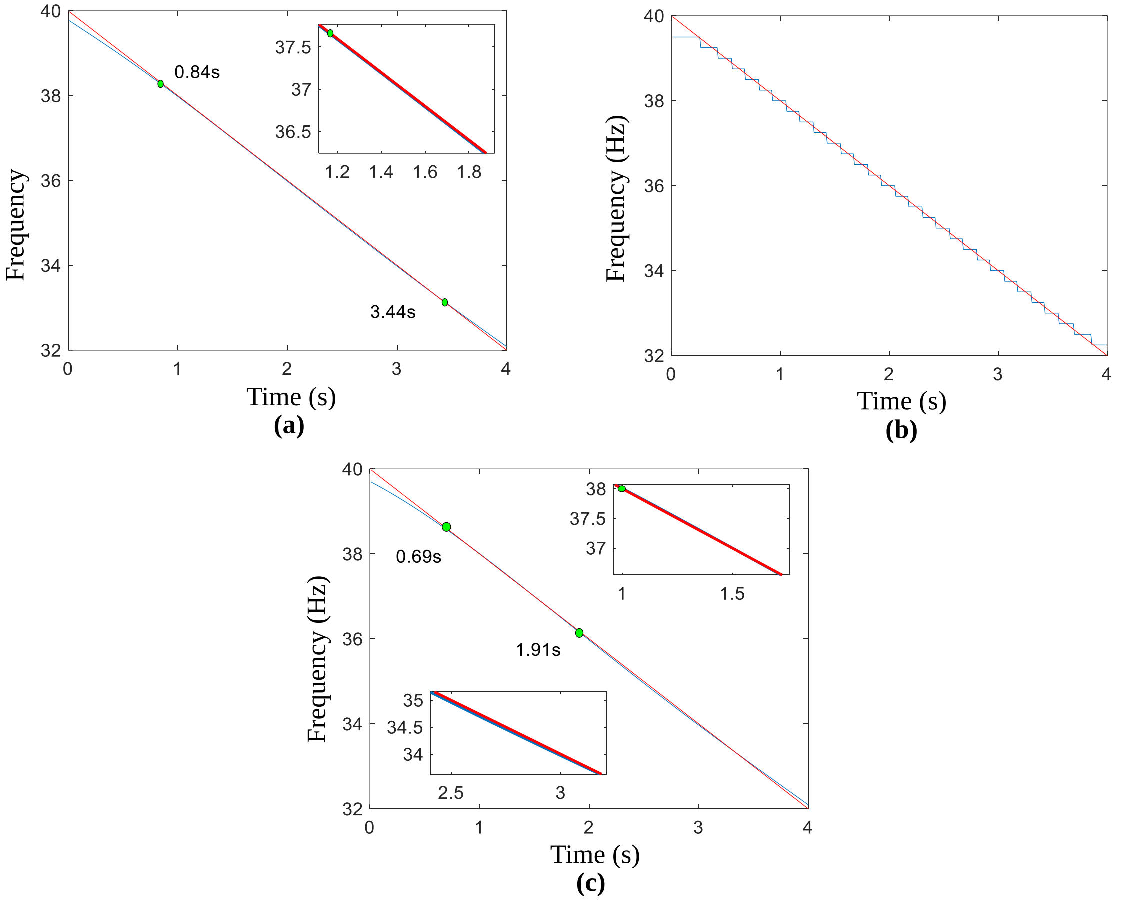

Fig. S1: Simulated signal. **(a)** Obtained result by using L2-based smooth method, **(b)** Obtained result by using L1-based smooth method with parameter 0.000001, **(c)** Obtained result by using polynomial curve fitting-based LSM method.

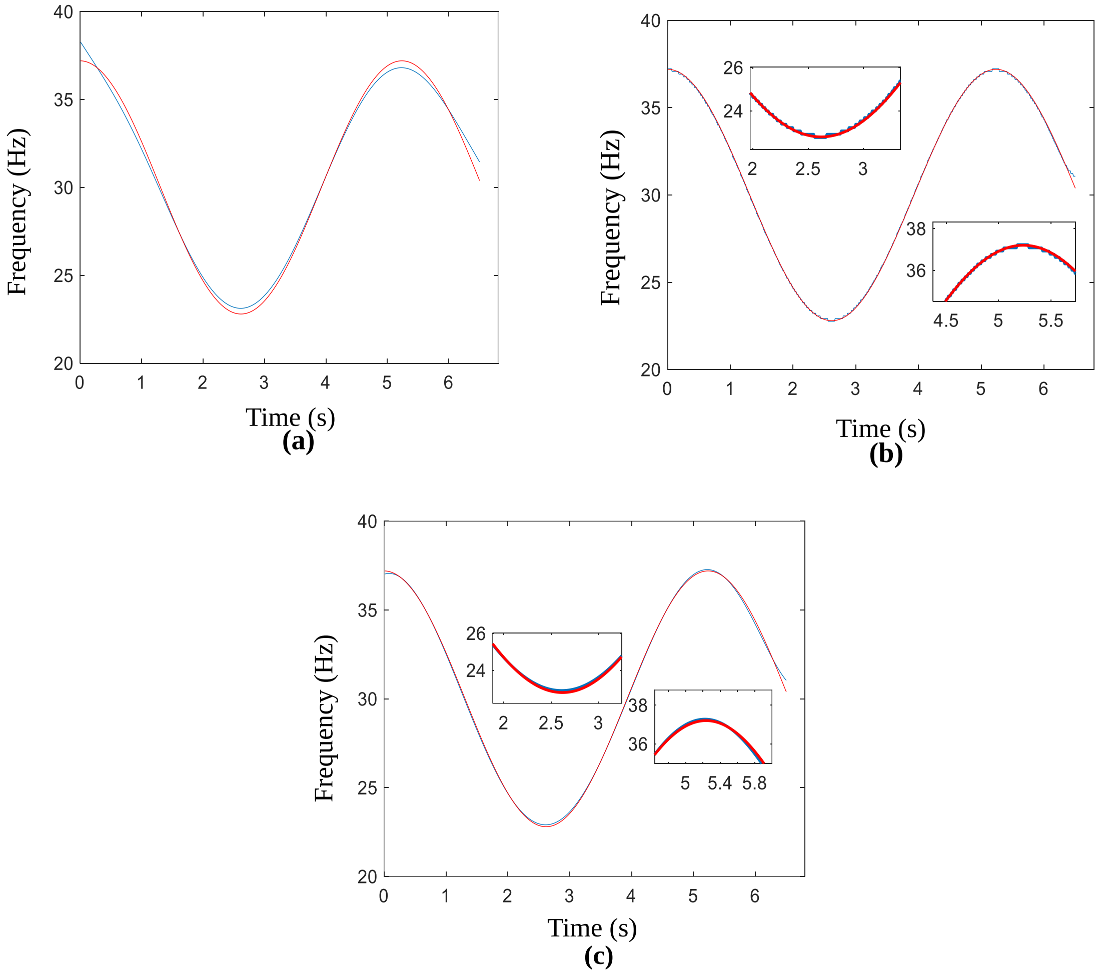

Fig. S3: Simulated signal. **(a)** Obtained result by using L2-based smooth method, **(b)** Obtained result by using L1-based smooth method with parameter 0.000001, **(c)** Obtained result by using polynomial curve fitting-based LSM method.

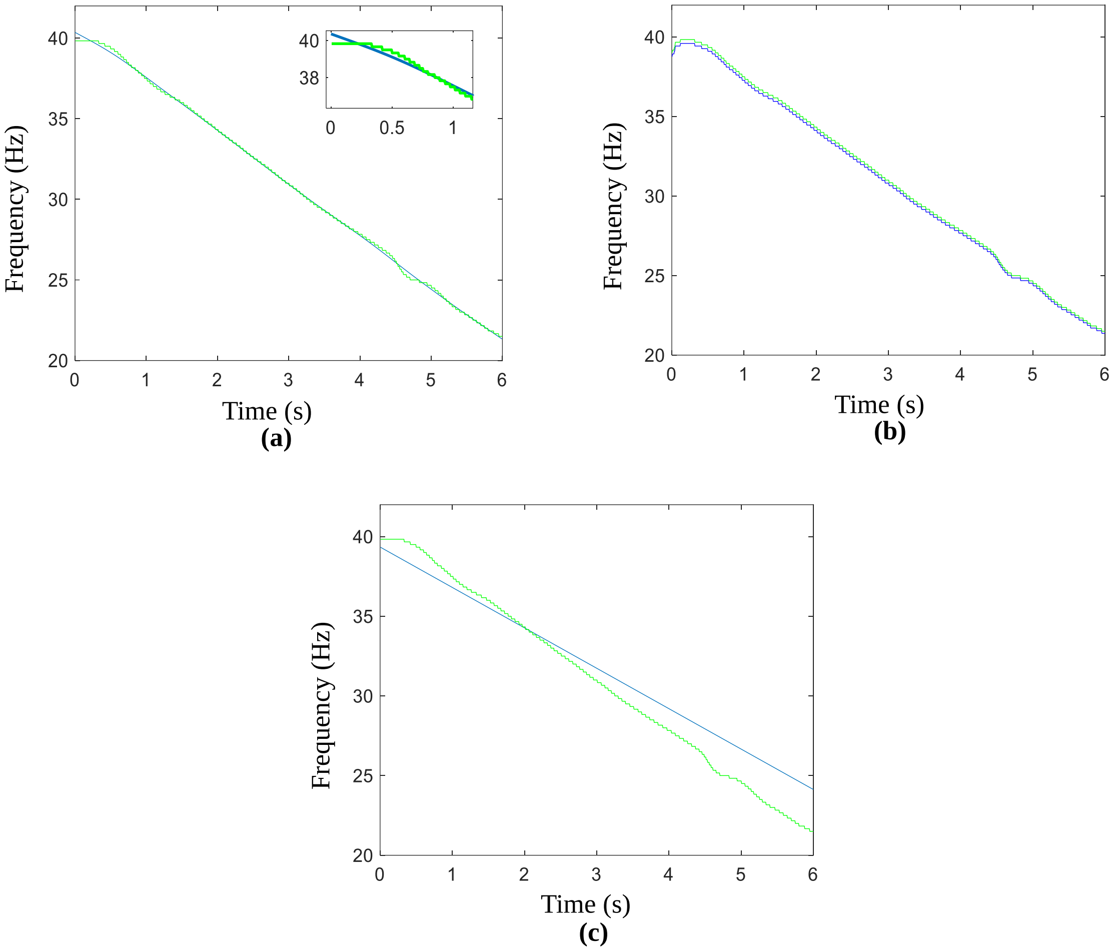

Fig. S5: Estimated and refined curve. **(a)** Obtained result by using L2-based smooth method, **(b)** Obtained result by using L1-based smooth method with parameter 0.000001, **(c)** Obtained result by using polynomial curve fitting-based LSM method.

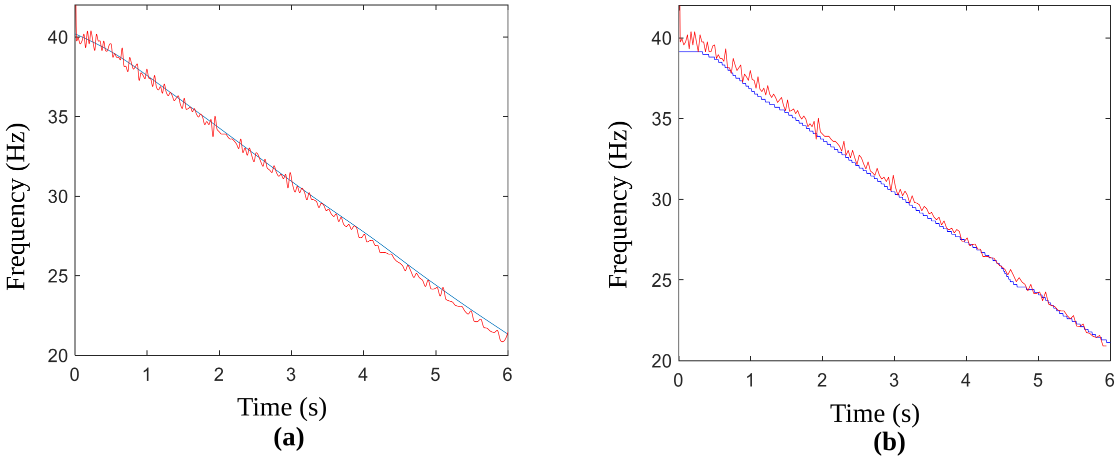

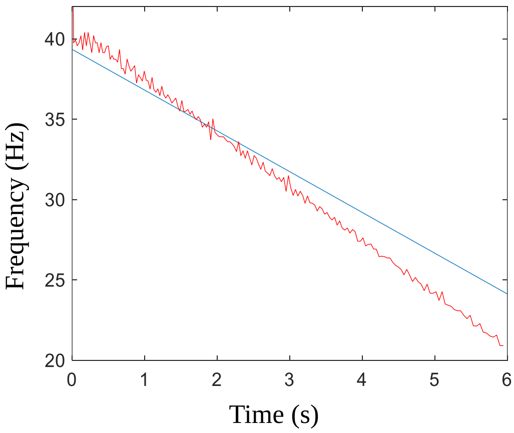

(c)

Fig. S6: Real and refined curve. **(a)** Obtained result by using L2-based smooth method, **(b)** Obtained result by using L1-based smooth method with parameter 0.000001, **(c)** Obtained result by using polynomial curve fitting-based LSM method.

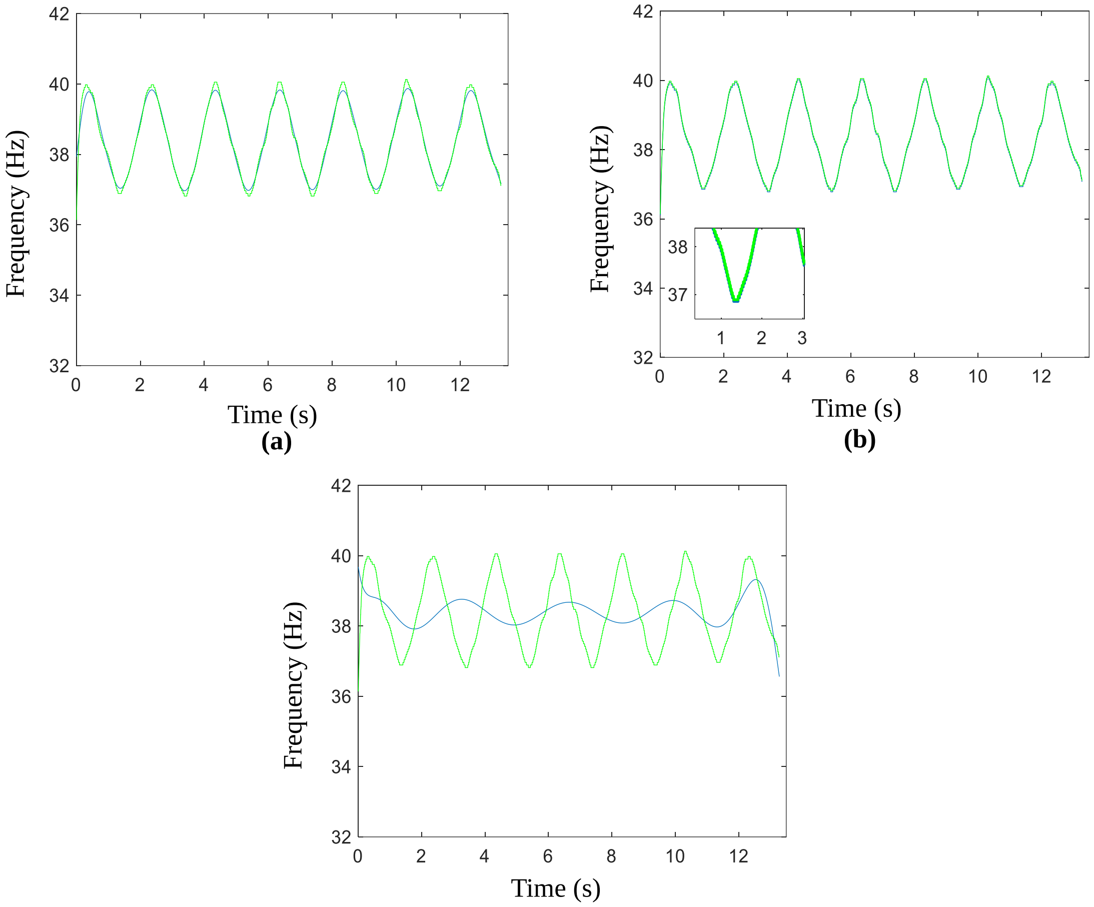

(c)

Fig. S7: Estimated and refined curve. **(a)** Obtained result by using L2-based smooth method, **(b)** Obtained result by using L1-based smooth method with parameter 0.000001, **(c)** Obtained result by using polynomial curve fitting-based LSM method.

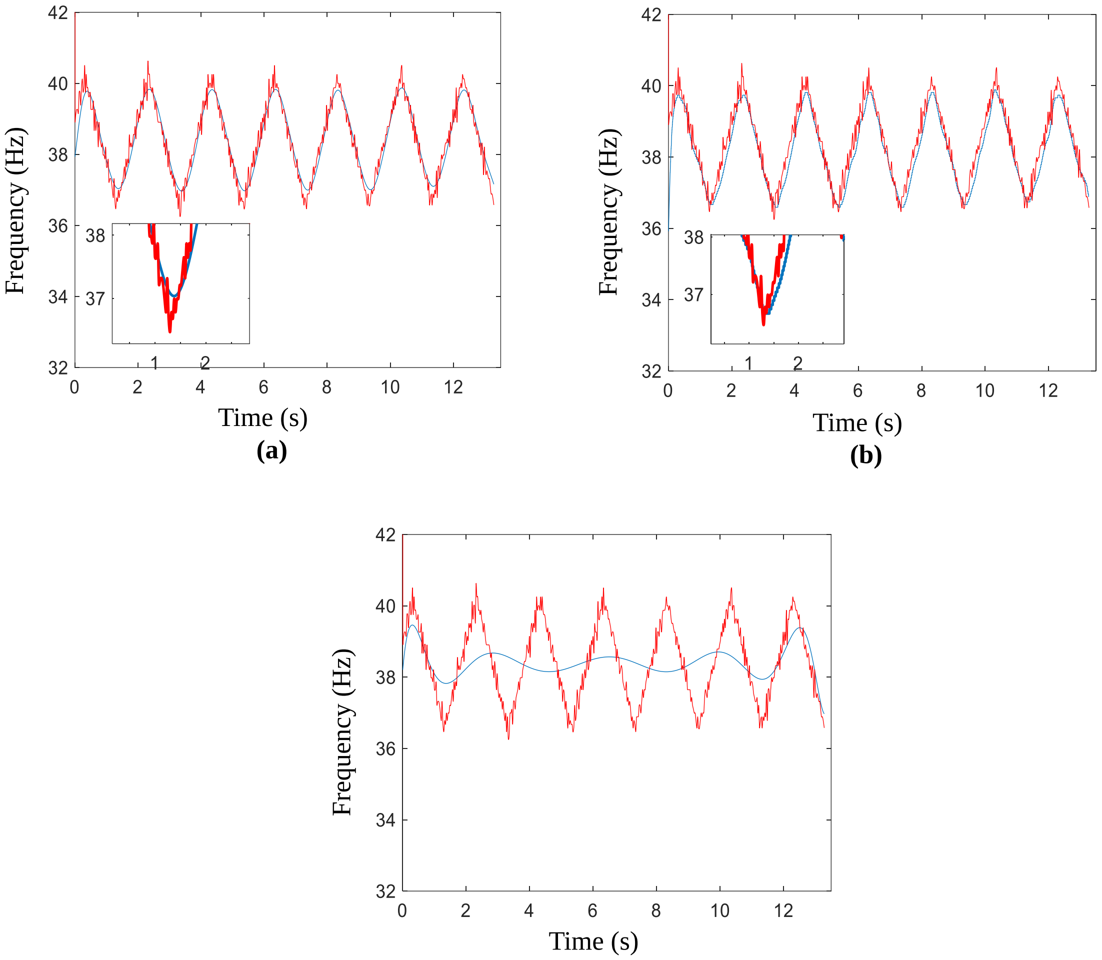

(c)

Fig. S8: Real and refined curve. **(a)** Obtained result by using L2-based smooth method, **(b)** Obtained result by using L1-based smooth method with parameter 0.000001, **(c)** Obtained result by using polynomial curve fitting-based LSM method.

Refined methods	L2-based	L1-based (0.000001)	AWMM	LSM
MAE	0.0392	0.0791	0.0411	0.0444

Table S1 MAE values of the four methods

Refined methods	L2-based	L1-based (0.000001)	LSM	AWMM
MAE	0.2776	0.0553	0.0743	0.0458

Table S3 MAE values of the four methods

Refined methods	L2-based	L1-based (0.000001)	AWMM
Estimated and refined	0.1038	0.3734	0.0436
Real and refined	0.2814	0.2644	0.2871

Table S5 MAE values of the three methods

Refined methods	L2-based	L1-based (0.0000002)	AWMM	LSM
Estimated and refined	0.0859	0.0375	0.0203	0.8347
Real and refined	0.2175	0.2053	0.2027	0.8566

Table S6 MAE values of the four methods
