# Supplementary material for: Refining the time–frequency characteristic of non-stationary signal for improving time–frequency representation under variable speeds
Source: Sci Rep. 2023 Mar 30;13:5215. doi: 10.1038/s41598-023-32333-w (PMC10063649; doi:10.1038/s41598-023-32333-w)
Supplement: Supplementary file 2 — Supplementary Information 2. [file 41598_2023_32333_MOESM2_ESM.pdf]

Considering that the experimental data provide the coarse curves, we develop the comparison cases of time-frequency analysis methods in simulation parts. We use traditional and enhanced time-frequency analysis methods to check the performance of AWSM, such as CWT and SST.

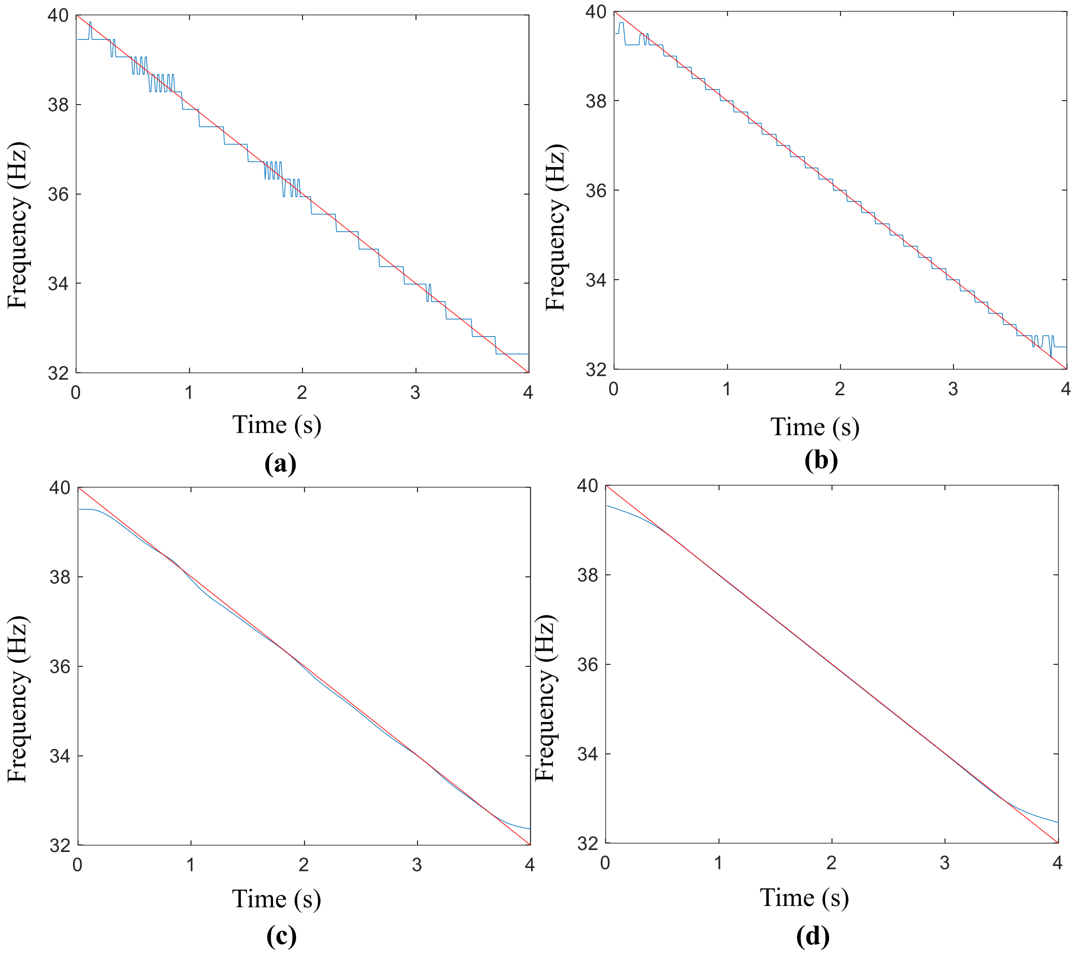

Fig. S2: Simulated signal. **(a)** Obtained result by using CWT, **(b)** obtained result by using SST, **(c)** the estimated curve by using CWT is smoothed, **(d)** the estimated curve by using SST is smoothed

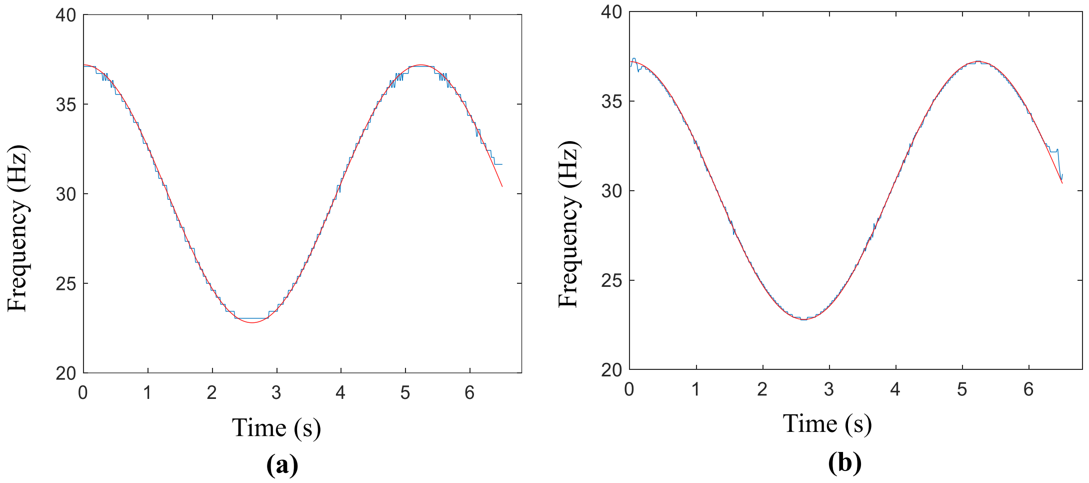

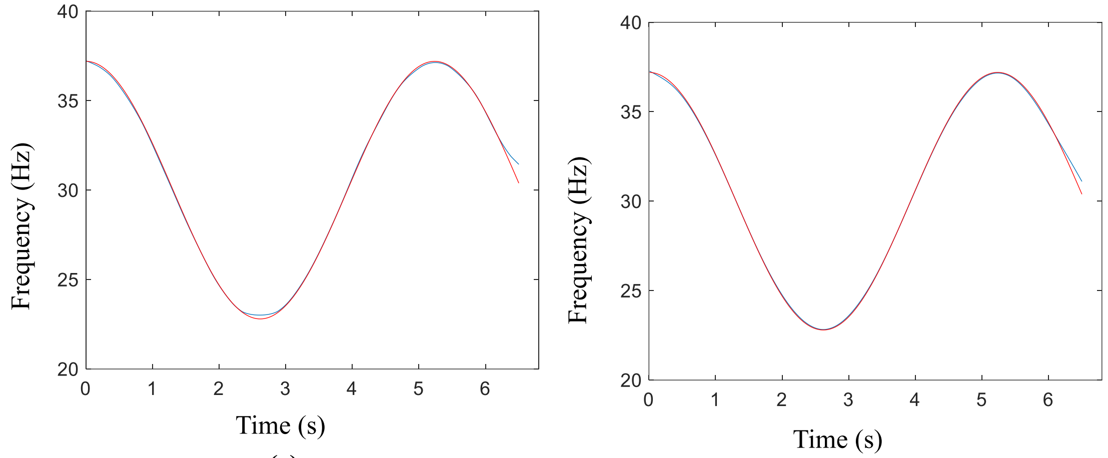

(c) **(d)**

Fig. S4: Simulated signal. **(a)** Obtained result by using CWT, **(b)** obtained result by using SST, **(c)** the estimated curve by using CWT is smoothed , **(d)** the estimated curve by using SST is smoothed

Time-frequency methods	MSST	CWT	SST
MAE	0.0411	0.0714	0.0544

Table S2 MAE values of the three time-frequency methods.

Time-frequency methods	MSST	CWT	SST
MAE	0.0458	0.0788	0.0667

Table S4 MAE values of the three time-frequency methods.
